# A New Case of Chanarin-Dorfman Syndrome with a Novel Deletion in *ABHD5* Gene

**DOI:** 10.29252/.22.6.415

**Published:** 2018-11

**Authors:** Shahrbanoo Nakhaei, Hamed Heidary, Aliasghar Rahimian, Mahdi Vafadar, Farzaneh Roohani, G.R. Bahoosh, Davoud Amirkashani

**Affiliations:** 1 Department of Pediatrics, Faculty of Medicine, Ali Asghar Children Hospital, Iran University of Medical Sciences (IUMS), Tehran, Iran; 2 Department of Medical Biochemistry, Tehran University of Medical Sciences, Tehran, Iran; 3 Pediatric Growth and Development Research Center, Iran University of Medical Sciences, Tehran, Iran

**Keywords:** Hepatomegaly, Ichthyosiform, Ichthyosis

## Abstract

Chanarin-Dorfman syndrome (CDS) is a rare autosomal recessive metabolic disorder caused by mutations in gene encoding the domain-5 of α/β-hydrolase enzyme (*ABHD5*). It is known as a natural lipid storage disorder arising from impaired lipid metabolism often characterized by hepatomegaly, myopathy, ataxia, non-bullous ichthyosiform erythroderma, hearing loss, and mental retardation. In the present study, we report two affected 28-month-old monozygotic twin boys as new cases of CDS. Genetic analysis was performed in patients, and the results showed a homozygote deletion in exon 4 of *ABHD5*. According to the the American College of Medical Genetics and Genomics, this variant is categorized as a pathogenic variant.

## INTRODUCTION

As a rare metabolic disorder, Chanarin-Dorfman syndrome (CDS, OMIM: #275630) arises from defective lipid metabolism caused by mutations in gene encoding α/β-hydrolase domain-5 (*ABHD5*)[1,2]. About 55 CDS cases has been reported so far, and the majority of them are from Mediterranean and Middle-Eastern countries, mainly Turkey[3,4].

Our study introduces two 28-month-old monozygotic twin boys with CDS. Clinical and laboratory findings include ichthyosis, elevated liver enzymes, hepatomegaly, and mutation in *ABHD5*. Liver fibrosis was detected by ultrasound imaging, and peripheral blood smear (PBS) revealed the Jordan’s anomaly in blood leucocytes. It should be noted that their parents are from two different villages with Arab origins (Hormozgan Province, Iran), and both are non-consanguineous.

### Case presentation

**Fig. 1:**
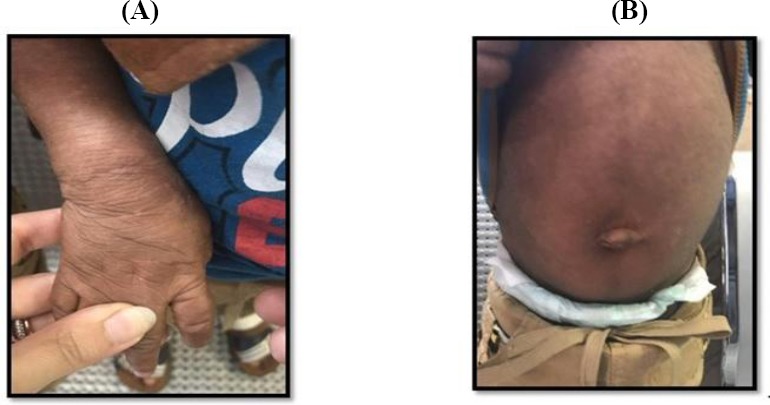
Clinical findings. Ichthyosis (A) and hepatomegaly (B).

The patients were two 28-month-old Iranian twin boys. They were referred to Ali Asghar Children’s Hospital (Tehran, Iran) with generalized dry and scaly skin along with itching-induced excoriations. They have had skin lesions since their birth as non-bullous erythrodermic ichthyosis without any hair, eye, and tooth complications. The severity of lesions had fluctuations, and the skin complications were only being treated with emollients (Fig. 1). At the age of six months, the patients had abdominal distention, based on their mother’s observation. Afterwards, numerous investigations had been carried out on metabolic and storage disorders, but they all had failed to draw any conclusion.

At the time of hospital admission, a significant ichthyosis and hepatomegaly (4 cm below rib edge in midclavicular line) was observed; however, no other signs and symptoms were detected. Growth and motor development milestones were normal according to the WHO standards[5]. Also, patients had normal muscular tone, and no eye and heart complications were detected. In primary investigations, heart, liver, and renal functions were normal. They only exhibited a mild increase in alanine transaminase (ALT) and aspartate transaminase (AST) levels, which were about 58 U/L and 63 U/L, respectively. Also, serum lipid profile for both patients was normal, except for triglyceride level, which was about 280 mg/dl.

Abdominal ultrasound showed a severe fatty infiltration of liver along with increased liver size without splenomegaly. In addition, previous metabolic investigations on PBS had shown obvious lipid vacuoles in cytoplasm of both neutrophils and monocytes. We aimed to perform a liver biopsy for better diagnosis but the parents refused consent.

### Differential diagnosis

**Fig. 2:**
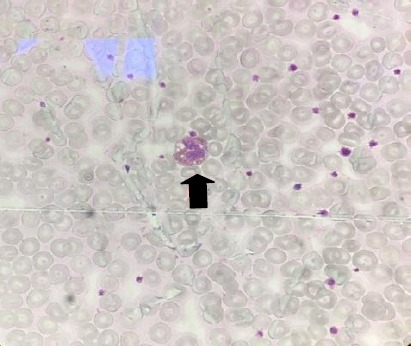
Peripheral blood smear. The arrow shows lipid vacuolization in leukocytes observed in blood smear (Jordan’s Anomaly).

Ichthyosiform erythroderma, as a usual symptom of CDS, is a typical manifestation in congenital ichthyosis syndromes, including Netherton, lamellar ichthyosis, epidermolytic hyperkeratosis, and Sjorgen-Larson syndrome, which are not lipid storage disorders. Therefore, PBS is necessary to examine for the presence of Jordan’s anomaly. In peripheral blood smears taken from our patients, lipid vacuolization was seen in the cytoplasm of leukocytes. This finding supports the presence of a natural lipid storage disorder (Fig. 2).

Defective carnitine metabolism is characterized by lipid storage in organs such as liver and muscle and consequent myopathy. However, this condition is simply differentiated from CDS by the absence of ichthyosis. In Wolman’s disease, acid lipase deficiency leads to defective lipid metabolism, resulting in a lysosomal storage disorder that manifests with massive hepatomegaly and failure to thrive. This syndrome is distinguished from CDS by the absence of ichthyosis. Also, renal calcifications and fatal outcome are known characteristics of Wolman’s disease, which are not seen in CDS. Multiple sulfatase deficiency is another metabolic disease sharing some clinical features with CDS, including ichthyosis and organomegalies. Coarse facial features and decreased activity of arylsulfatases differentiate multiple sulfatase deficiency from CDS[6].

### Genetic studies

#### DNA extraction

Genomic DNA was extracted from the blood leucocytes of the proband using PrimePrep™ G enomic DNA Eextraction Kit (GeNet Bio, South Korea) according to the instructions provided by the manufacturer.

#### Polymerase chain reactions (PCR)

Mutation analysis was performed in affected patients. Initially, all 7 exons of *ABHD5* (NM_016006) were amplified using PCR method. The primer sequences are shown in Table 1. PCR reactions were performed in LabCycler (SensoQuest, Germany) using 2× PCR Master Mix (Pishgam Biotech Co., Tehran, Iran). The volume of PCR reactions was 40 μl containing 100 ng of genomic DNA and primers at a concentration of 270 nM (for each forward and reverse primer). PCR started at 95 °C for 3 min, followed by 35 cycles of 95 °C for 30 s, 59 °C for 30 s, and 72 °C for 1 min, with the final extension at 72 °C for 7 min. Afterwards, PCR products for all 7 exons were purified and sequenced on an ABI PRISM *373XL sequencer* (Macrogen, South *Korea)*.

**Table 1:** Primers used in this study

*ABHD5* exons	Sequence		Amplicon size (bp)

	Forward (5'→')	Reverse (5'→')	
1	TAAAACACCTAACTCATTCGGG	TTATACAACAACGGGGCGGAC	641
2	CCATTCTTTGTGCATGTTAG	AAACAAATCTCCTTGGGGTC	492
3	ACTTGTCCTTCTCCATGGTTTTG	CCTTGATGGGTACTTCAGGGAG	277
4	TACACCTCTTAGATCCTACTGA	GACTAACCTTATCCTCAGCATTC	611
5	CACAGACAAGCACTAAAACTTTC	GACCTGGGGTCAGAAGTTCA	545
6	CTTAGGTGCTGGAAAAGCTA	GTAGTTCACGGTTTGGACAT	492
7	TTTAAATACAGTGGCTCTCACTT	TCAGAAATCACTTCCTAAATTGG	572

## RESULTS

**Fig. 3:**
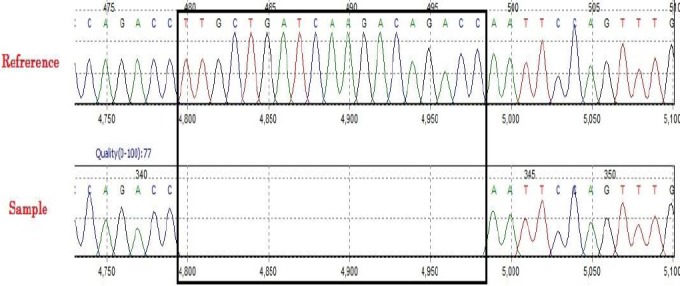
The DNA sequence for exon 4 of *ABHD5*. A 19-bp deletion in DNA samples was observed in patients compared with a reference DNA. The box shows comparison of genome reference and case sequence in the deleted sequence.

The results of DNA sequencing for *ABHD5* exons showed no differences between patients and genomic DNA reference for exons 1, 2, 3, 5, 6, and 7. However, as for exon 4, a homozygote deletion (c. 560_578 delTTGCTGATCAAGACAGACC) was detected (Fig. 3). The mutation result showed L187Qfs*13 using MutationTaster database (http://www.mutationtaster.org), and more investigations were done for pathogenecity. This variant was neither found in ExAC (exac.broadinstitute.org) nor in 1000G (www.internationalgenome.org) databases, and it caused a frameshift, eventually leading to a nonsense-mediated mRNA decay (NMD). Based on the MutationTaster database, this variant causes CDS due to impaired α/β-hydrolase activity. It should be noted that the twin patients had the same genetic test results.

We prescribed 25 mg/kg/day Neotigason to treat skin lesions. Also, medium-chain triglycerides oil and partial fat restriction diet were included in treatment plan. Interestingly, after a six-month treatment, the skin lesions improved and the hepatomegaly subsided (1 cm below rib edge in midclavicular line) without any observable impairment in hepatic function. Besides, no more reduction in liver size was achieved afterwards.

## DISCUSSION

CDS is a rare lipid storage disorder characterized by ataxia, non-bullous ichthyosiform erythroderma, hearing loss, and mental retardation, and it is usually associated with hepatomegaly and myopathy. Clinical symptoms arise from the storage of excess triglycerides in multiple tissues due to defective triacylglycerol catabolism[7,8]. Defects in *ABHD5* often lead to ichthyosis[9]. As the most noticeable finding of the CDS, skin complications may be the consequence of a deformity in the permeability barrier of the skin due to lipid storage disorder. In fact, errors in *ABHD5* function result in impaired triglyceride degradation, giving rise to non-polar triglyceride inclusions in the lamellar bodies as the final outcome[9-11].

CDS diagnosis is based on clinical findings and the presence of lipid vacuoles in granulocytes in Giemsa staining of PBS, known as Jordan’s anomaly[12,13]. *ABHD5* is broadly expressed in different tissues, including skeletal muscle, lymphocytes, skin, liver, and brain. Therefore, any impairment in *ABDH5* expression may give rise to a wide spectrum of complications involving various tissues and organs[14].

The present study introduces two 28-month-old monozygotic twin boys with skin lesions as the chief complaint. No other signs and symptoms were detected in physical examinations, except organomegaly that was confirmed by sonography as hepatomegaly without splenomegaly. PBS showed lipid vacuoles in polymorphonuclear cells and monocytes. All the metabolic investigations were normal. Liver biopsy seems not to be necessary for diagnosis, but it may be beneficial for evaluation of response to treatments with Neotigason and lipid-restricting diets.

Analysis of coding region by sequencing method showed a novel mutation (deletion in exon 4) in *ABHD5* and according to MutationTaster, this variant can lead to NMD. As a natural surveillance mechanism to eliminate mutant mRNAs, NMD degrades the mRNAs harboring premature termination codons[15]. According to the joint consensus recommendation of the American College of Medical Genetics and Genomics and the Association for Molecular Pathology, for the interpretation of sequence variants, the strategies used for the analysis of the novel variant in this study effectively showed its pathogenicity[16], which includes: (I) the absence of the variant in population databases makes a moderate evidence for pathogenicity (PM2), (II) the clinical and laboratory findings in proband patients, who were highly specific for CDS due to defective *ABHD5*, provides a supporting evidence for pathogenicity (PP4), (III) multiple lines of computational evidence, indicating a deleterious effect on the gene function, made a supporting evidence for the pathogenicity (PP3), and (IV) predicted null variant in a gene, where loss of function is a known mechanism of disease, provides a very strong evidence for pathogenicity (PVS1). In conclusion, due to one very strong, one moderate, and two supporting evidence for the pathogenicity of this variant, it can be considered as a pathogenic variant.
